# Intraoperative Corneal Thickness Changes during Pulsed Accelerated Corneal Cross-Linking Using Isotonic Riboflavin with HPMC

**DOI:** 10.1155/2016/1471807

**Published:** 2016-01-13

**Authors:** Ahmed M. Sherif, Nihal A. El-Gheriany, Yehia M. Salah El-Din, Lamiaa S. Aly, Amr A. Osman, Michael A. Grentzelos, George D. Kymionis

**Affiliations:** ^1^Department of Ophthalmology, Faculty of Medicine, Cairo University, Cairo 11519, Egypt; ^2^Vardinoyiannion Eye Institute of Crete (VEIC), Faculty of Medicine, University of Crete, Heraklion, 71003 Crete, Greece; ^3^Bascom Palmer Eye Institute, University of Miami, Miller School of Medicine, Miami, FL 33136, USA

## Abstract

*Purpose*. To evaluate corneal thickness changes during pulsed accelerated corneal cross-linking (CXL) for keratoconus using a new isotonic riboflavin formula.* Methods*. In this prospective, interventional, clinical study patients with grades 1-2 keratoconus (Amsler-Krumeich classification) underwent pulsed accelerated (30 mW/cm^2^) CXL after application of an isotonic riboflavin solution (0.1%) with HPMC for 10 minutes. Central corneal thickness (CCT) measurements were taken using ultrasound pachymetry before and after epithelial removal, after riboflavin soaking, and immediately after completion of UVA treatment.* Results*. Twenty eyes of 11 patients (4 males, 7 females) were enrolled. Mean patient age was 26 ± 3 (range from 18 to 30 years). No intraoperative or postoperative complications were observed in any of the patients. Mean CCT was 507 ± 35 *μ*m (range: 559–459 *μ*m) before and 475 ± 40 *μ*m (range: 535–420 *μ*m) after epithelial removal (*P* < 0.001). After 10 minutes of riboflavin instillation, there was a statistically significant decrease of CCT by 6.2% from 475 ± 40 *μ*m (range: 535–420 *μ*m) to 446 ± 31 *μ*m (range: 508–400) (*P* < 0.005). There was no other statistically significant change of CCT during UVA irradiation.* Conclusions*. A significant decrease of corneal thickness was demonstrated during the isotonic riboflavin with HPMC application while there was no significant change during the pulsed accelerated UVA irradiation.

## 1. Introduction

Corneal cross-linking (CXL) is a minimally invasive procedure that combines the use of riboflavin and ultraviolet-A (UVA) irradiation resulting in an increase of the biomechanical stability of the corneal tissue [1, 2]. A preoperative corneal thickness of 400 *μ*m as a minimum safety limit to avoid corneal endothelial damage during CXL has been proposed [3]. However, endothelial failure has been reported very occasionally after CXL resulting in corneal edema postoperatively [4, 5]. The etiology of such problems has not been fully elucidated but may be due to severe stromal thinning intraoperatively which has been reported by several authors [6, 7]. Hence, it has become important to monitor corneal thickness during the procedure.

Accelerated CXL is based on the Bunsen-Roscoe law of reciprocity according to which reducing irradiation time and correspondingly increasing irradiation intensity could achieve the same photochemical effect.

The aim of this study was to evaluate the intraoperative pachymetric changes during CXL using isotonic riboflavin (0.1%) and HPMC (hydroxyl propyl methylcellulose, HPMC; VibeX Rapid, Avedro Inc., Waltham, MS, USA) and pulsed accelerated UVA.

## 2. Materials and Methods

In this prospective, interventional, clinical study patients with grades 1-2 keratoconus (Amsler-Krumeich classification) were enrolled. All patients underwent pulsed high intensity CXL using the KXL system (Avedro Inc., Waltham, MS, USA) preceded by the application of an isotonic riboflavin (0.1%) and HPMC (hydroxyl propyl methylcellulose, HPMC; VibeX Rapid, Avedro Inc., Waltham, MS, USA) for 10 minutes at Eye Care Center, Maadi, Cairo, Egypt, between August 2014 and February 2015. The study was conducted within the tenets of the Declaration of Helsinki after obtaining the institutional review board approval. A written informed consent was obtained from all patients.

Inclusion criteria were progressive keratoconus (progression was confirmed if there was an increase in the *K*_max_ on Pentacam maps of 1.00 diopter [D], increase of manifest refraction cylinder of 1.00 D, or increase of manifest refraction spherical equivalent of 0.50 D over the period of one year) and corneal thickness more than 400 *μ*m at the thinnest location. Exclusion criteria were corneal scars or opacities, pregnancy or lactation, active anterior segment pathologic features, previous corneal or anterior segment surgery, systemic connective tissue disease, atopic syndrome, and dry eye syndrome. Preoperative data obtained from the case records included patient age and gender, Pentacam central corneal thickness (CCT) and thinnest corneal thickness (TCT) values, and CCT values obtained by ultrasonic corneal pachymetry (Sonomed 300P PacScan Pachymeter; Escalon Medical Corp.), which takes the mean of 256 measurements in each scan.

### 2.1. Surgical Technique

Corneal cross-linking (CXL) was conducted under sterile conditions. One drop of pilocarpine 1% eye drops was instilled 15 minutes before the procedure. After topical application of benoxinate hydrochloride 0.4% eye drops (Benox; Eipico Inc., Cairo, Egypt), an eye speculum was placed and CCT was measured just before epithelial removal. The probe tip of the ultrasonic pachymetry was disinfected using alcohol swab and was held perpendicular to the cornea. Three consecutive measurements were obtained at the center of the cornea of each eye; the thinnest measurement is used in the statistical analysis. Then, the central 8 mm of the corneal epithelium was removed mechanically using a blunt spatula. After corneal epithelial removal, CCT was measured. Next, dextran-free riboflavin 0.1% with hydroxyl propyl methylcellulose (HPMC; VibeX Rapid, Avedro Inc., Waltham, MS, USA) was instilled every 2 minutes for 10 minutes after which CCT was remeasured. Pulsed accelerated UVA irradiation was next performed using KXL system (Avedro Inc., Waltham, MS, USA) with 1 sec. on/1 sec. off of UVA irradiation of 30 mW/cm^2^ for a total duration of 8 minutes and 40 seconds. A final CCT measurement was obtained immediately after completion of UVA irradiation. A therapeutic contact lens was applied and removed at the 3rd postoperative day after complete reepithelialization.

### 2.2. Statistical Analysis

Statistical analysis was done using paired *t*-test. Statistical Package for the Social Sciences (SPSS) v.16 was used.

Because both eyes of some patients were used in the study, a nested analysis of variance was used to correct for any correlation between the right and left eyes of the same subject. *P* value less than 0.05 was considered significant.

## 3. Results

Twenty eyes of 11 patients were included. Four were males and 7 were females. Mean patient age was 26 ± 3 (range from 18 to 30 years). No intraoperative or postoperative complications were observed in any of the patients.

**Figure 1:**
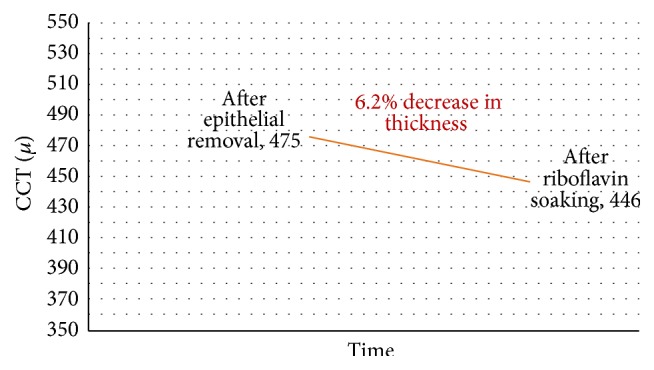
CCT changes (in microns) after riboflavin soaking.

**Figure 2:**
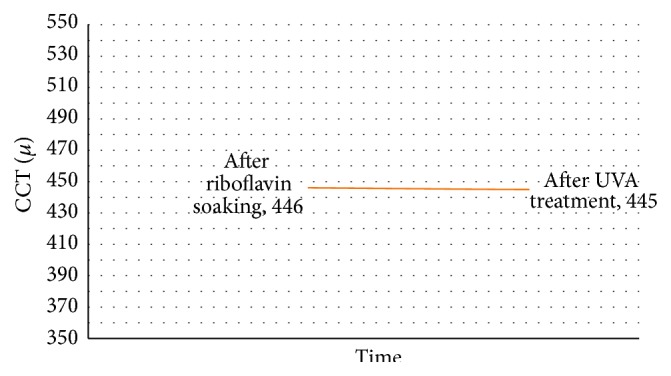
CCT changes (in microns) during UVA treatment.

Mean USP CCT was 506.85 ± 35.4 *μ*m (range: 559–459 *μ*m) before and 474.9 ± 39.75 *μ*m (range: 535–420 *μ*m) after epithelial removal (*P* < 0.001). After 10 minutes of riboflavin installation, there was a statistically significant decrease of CCT by 6.2% (31.95 ± 8.08 *μ*m) from 474.9 ± 39.75 *μ*m (range: 535–420 *μ*m) to 445.7 ± 31.18 *μ*m (range: 508–400 *μ*m) (*P* < 0.005) (Figure 1). There was no statistically significant change of the CCT during UVA irradiation; CCT was 444.65 ± 34.53 *μ*m (range: 521–400 *μ*m) at the end of UVA irradiation (*P* = 0.61) (Figure 2). However, there was a statistically significant decrease by 62.2 ± 34.64 *μ*m in CCT from 506.85 ± 35.4 *μ*m (range: 559–459 *μ*m) before epithelial removal to 444.65 ± 34.53 *μ*m (range: 521–400 *μ*m) at the end of CXL (*P* < 0.001). Results are summarized in Table 1.

## 4. Discussion

Several studies have already reported the intraoperative corneal thickness changes during the CXL procedure using several riboflavin formulas and different UVA settings. Most of the studies evaluated the classic Dresden protocol according to which riboflavin-dextran solution was applied for 30 minutes followed by 30 minutes of UVA 3 mW/cm^2^. Thus, Kymionis et al. reported a 20% decrease in CCT after 30 minutes of riboflavin instillation [6]. Mazzotta and Caragiuli observed 32% decrease after 30 minutes of riboflavin-dextran solution instillation; at 10 minutes of the 30-minute instillation procedure the CCT decrease was almost 18% [7]. Hassan et al. showed 23.76% decrease after 15 minutes of riboflavin application [8]. They all reported no corneal thickness changes during UVA treatment [6–8]. However, Tahzib et al. showed stable CCT values after riboflavin solution instillation, which might be attributed to the fact that they removed the eye speculum during riboflavin application [9]. Soeters et al. indicated that avoidance of an eyelid speculum during riboflavin instillation resulted in less CCT reduction [10].

The use of isotonic riboflavin solution without dextran has been suggested to avoid the dehydrating effect of dextran on the corneal stroma. Çinar et al. showed swelling of the cornea instead of corneal thinning after 30 minutes of isotonic riboflavin solution without dextran during CXL procedure [11]. In a comparative study, Oltulu et al. evaluated the corneal thickness changes during CXL performed with isotonic riboflavin solution with and without dextran; the use of riboflavin solution without dextran during CXL caused a steady increase in the corneal thickness during the procedure [12]. Recently, Jain et al. demonstrated no significant decline in the corneal thickness after CXL using isotonic riboflavin with HPMC with accelerated CXL [13].

In this clinical study, an isotonic dextran-free riboflavin formula with HPMC (HPMC; VibeX Rapid, Avedro Inc., Waltham, MS, USA) was used in combination with pulsed accelerated UVA using the KXL system (Avedro Inc., Waltham, MS, USA). The addition of HPMC to 0.1% riboflavin aims to avoid the dehydrating effect of dextran while maintaining adequate concentration of riboflavin into the stroma through its viscosity. After 10 minutes of riboflavin solution instillation, there was a statistically significant decrease of CCT by 6.2% (31.95 ± 8.08 *μ*m) from 475 ± 40 *μ*m (range: 535–420 *μ*m) to 446 ± 31 *μ*m (range: 508–400 *μ*m). There was no other statistically significant change of the CCT during UVA irradiation.

It seems that there was a lesser reduction of CCT in our study compared to previous studies in which riboflavin with dextran was used [7–9]. In the study of Jain et al., no significant difference was found after 20 minutes of isotonic riboflavin with HPMC [13]. However, they did not perform pachymetry measurement at 10 minutes of the 20-minute UVA irradiation process [13]. In our study, we performed a 10-minute riboflavin instillation process and a 6.2% CCT decrease was measured after 10 minutes of isotonic riboflavin with HPMC instillation. In any case, it seems that isotonic riboflavin with HPMC may lead to a significant decrease in the corneal thickness during the instillation process of the CXL procedure, but less than the decrease reported in previous studies using riboflavin-dextran solution [6–8]. Therefore, this riboflavin formula might be actually safer for CXL especially for keratoconic patients with borderline preoperative corneal thickness (near to 400 *μ*m).

Potential limitations of the study included the small sample size, the fact that repeated central corneal thickness, measured by ultrasound pachymetry, is strongly affected by exact centration of the probe on cornea on each measurement, and the fact that the gradient of thickness variation is strong particularly in keratoconic corneas. Taking into consideration that each scan with the Sonomed 300P pachymeter takes the mean of 256 measurements, 3 scans were performed with the thinnest measurement (the lowest mean of 256 measurements) in an attempt to respect the safety aspect during planning or conduction of the procedure.

Pentacam or AS-OCT is much better for corneal thickness assessment than ultrasound pachymetry, especially when a two-dimensional surface is to be analyzed for the thinnest point, but in our study, it was not possible to use the Pentacam intraoperatively for measurements after epithelial removal, after riboflavin, and after UVA exposure as the procedure was performed under aseptic techniques in the operation theater while the Pentacam device is not portable. Repeated transfer of the patient between the operating theater and the investigation unit was judged not feasible due to patient discomfort, the potential risk of contamination, and corneal dehydration.

Preoperative Pentacam CCT values were recorded but were not used instead of the preoperative contact pachymetry CCT values to avoid the bias of using one measurement device preoperatively and another type of measurement device intraoperatively.

In conclusion, a significant corneal pachymetric decrease was demonstrated after 10 minutes of isotonic riboflavin with HPMC instillation during pulsed accelerated CXL. This decrement was less than the reduction observed in previous studies using riboflavin-dextran solution [7–9]. There was no corneal thickness change during pulsed accelerated UVA irradiation. These findings suggest that isotonic riboflavin with HPMC might be useful in CXL treatment of keratoconic patients with thin corneas. Further randomized controlled trials with larger sample sizes are needed to validate these observations.

## Figures and Tables

**Table 1:** Summary of central epithelial thickness changes.

CCT in microns	Mean	Standard deviation	Maximum	Minimum
Preoperative Pentacam CCT	514.9	±33.12	561	470
Preoperative USP CCT	506.85	±35.4	559	459
USP CCT after epithelial removal	474.9	±39.75	535	420
USP CCT after riboflavin	445.7	±31.18	508	400
USP CCT after UVA	444.65	±34.53	521	400

CCT: central corneal thickness, USP: ultrasound pachymetry, and UVA: ultraviolet.
